# GUT MICROBIOTA AND THE USE OF PROBIOTICS IN CONSTIPATION IN CHILDREN AND ADOLESCENTS: SYSTEMATIC REVIEW

**DOI:** 10.1590/1984-0462/2020/38/2018123

**Published:** 2019-11-25

**Authors:** Daiane Oliveira Vale San Gomes, Mauro Batista de Morais

**Affiliations:** a Universidade Federal de São Paulo, São Paulo, SP, Brazil.

**Keywords:** Constipation, Probiotics, Microbiota, Child, Adolescent, Constipação intestinal, Probiótico, Microbiota, Criança, Adolescente

## Abstract

**Objective:**

To perform a systematic review of literature data on gut microbiota and the efficacy of probiotics for the treatment of constipation in children and adolescents.

**Data source:**

The research was performed in the PubMed, the Scientific Electronic Library Online (SciELO) and the Latin American and Caribbean Health Sciences Literature (LILACS) databases in English, Portuguese and Spanish. All original articles that mentioned the evaluation of the gut microbiota or the use of probiotics in children with constipation in their title and abstract were selected.

**Data synthesis:**

559 articles were found, 47 of which were selected for reading. From these, 12 articles were included; they studied children and adolescents divided into two categories: a gut microbiota evaluation (n=4) and an evaluation of the use of probiotics in constipation therapy (n=8). The four papers that analyzed fecal microbiota used different laboratory methodologies. No typical pattern of gut microbiota was found. Regarding treatment, eight clinical trials with heterogeneous methodologies were found. Fifteen strains of probiotics were evaluated and only one was analyzed in more than one article. Irregular beneficial effects of probiotics have been demonstrated in some manifestations of constipation (bowel frequency or consistency of stool or abdominal pain or pain during a bowel movement or flatulence). In one clinical trial, a complete control of constipation without the use of laxatives was obtained.

**Conclusions:**

There is no specific pattern of fecal microbiota abnormalities in constipation. Despite the probiotics’ positive effects on certain characteristics of the intestinal habitat, there is still no evidence to recommend it in the treatment of constipation in pediatrics.

## RESUMO

**Objetivo:**

Realizar revisão sistemática dos dados da literatura sobre a microbiota intestinal e a eficácia dos probióticos para o tratamento da constipação intestinal em crianças e adolescentes.

**Fonte de dados:**

Foi realizada busca nas bases de dados PubMed, Scientific Electronic Library Online (SciELO) e Literatura Latino-Americana e do Caribe em Ciências da Saúde (LILACS), em inglês, português e espanhol. Foram selecionados, pelo título e pelo resumo, todos os artigos originais que avaliaram a microbiota intestinal ou o emprego de probióticos em crianças com constipação intestinal.

**Síntese dos dados:**

Foram encontrados 559 artigos, dos quais 47 foram selecionados para leitura. Destes, foram incluídos 12 artigos que estudaram crianças e adolescentes distribuídos em duas categorias: avaliação da microbiota intestinal (n=4) e avaliação do emprego dos probióticos na terapêutica da constipação intestinal (n=8). Os quatro artigos que analisaram a microbiota fecal utilizaram metodologias laboratoriais diferentes. Não foi observado um padrão típico de microbiota intestinal. Quanto ao tratamento, foram encontrados oito ensaios clínicos com metodologias heterogêneas. Foram avaliadas 15 cepas de probióticos e apenas uma foi avaliada em mais de um artigo. Foram evidenciados efeitos benéficos não uniformes dos probióticos em algumas manifestações da constipação intestinal (frequência evacuatória, consistência das fezes, dor abdominal, dor ao evacuar ou flatulência). Em apenas um ensaio clínico foi obtido completo controle da constipação intestinal sem o emprego concomitante de laxantes.

**Conclusões:**

Não existe um padrão específico de anormalidades da microbiota fecal na constipação intestinal. Apesar dos efeitos positivos dos probióticos em determinadas características do hábito intestinal, ainda não existem evidências que permitam sua recomendação no tratamento da constipação intestinal em pediatria.

## INTRODUCTION

Constipation is a common clinical occurrence in children and adolescents, with over 90% of cases that classify as functional gastrointestinal disorders.^1^ ^,^ ^2^ The prevalence of constipation varies depending on the diagnostic criteria used, and it is considered to be a public health problem.^3^ The Rome criteria are currently adopted to standardize the diagnosis of constipation in the pediatric population.^4^ ^,^ ^5^

An important role of retention behavior has been evidenced in the pathophysiology of functional constipation, due to unpleasant experiences with bowel movements. Recommended treatment includes a combination of dietary interventions (adequate fiber and fluid intake), education, demystification and, where appropriate, toilet training and completion of a bowel movement diary. In the presence of fecaloma, fecal disimpaction and the use of oral laxatives for prolonged treatment are indicated. The effectiveness and safety of these procedures are well established.^1^ ^,^ ^5^ ^,^ ^6^

However, for a small portion of patients, conventional treatment does not provide satisfactory improvement, which leads to interest in other therapeutic strategies. There is also a great deal of concern from family members about the prolonged use of laxatives.^1^ ^.^ ^7^

It is suggested that prolonged fecal stasis in the colon of patients with constipation has an impact on gut microbiota, which may influence various intestinal functions, including motility.^8^ ^,^ ^9^ In adults with constipation, the fecal microbiota was found to be different from healthy controls, however the results are heterogeneous.^10^ ^,^ ^11^ ^,^ ^12^ A study conducted in Ireland in 2005 showed a decrease in the abundance of *Bifidobacterium* and *Lactobacillus*.^10^ In 2015, a clinical trial conducted in Korea also found a decrease of *Bifidobacterium* spp., and reported a decrease in abundance of the *Bacteroides* species, when compared to the control group.^11^

In contrast, in 2016, it was observed in the colonic and fecal mucosa of women with intestinal constipation, that the fecal microbiota profile was not associated with increased colonic transit time, when confounding factors such as age, body mass index and diet were considered. On the other hand, *Faecalibacterium*, *Lactococcus* and *Roseburi*, which are genus of the phylum *Firmicutes,* correlated with decreased colonic transit.^12^

The abnormalities observed in the gut microbiota in patients with constipation have increased the use of probiotics for additional support in their treatment.^11^ ^.^ ^13^ In addition, they are probably more accepted by families than traditionally used laxatives. As such, this systematic review of literature data on gut microbiota and the efficacy of using probiotics for the treatment of constipation in children and adolescents was performed.

## METHOD

This review article was conducted according to the PRISMA criteria for systematic reviews and meta-analyzes.^14^ The PubMed, the Scientific Electronic Library Online (SciELO), and the Latin American and Caribbean Health Sciences Literature (LILACS) databases were used to search the literature. A study was conducted on articles in English, Portuguese and Spanish that analyzed the microbiota profile and the use of probiotics in the treatment of constipation in children and adolescents. In PubMed, the period from January 1966 to December 31, 2017 was evaluated. To this end, a broad search was performed using the following terms and operators: (gastrointestinal microbiome OR microbiome OR microbiota OR ecosystem) OR (probiotic OR *bifidobacterium* OR *lactobacillus*) AND constipation. In the LILACS and SciELO databases, no research period was defined. The oldest record in each one up until the last one from December 2017 was considered. The following terms and limits were used: (gastrointestinal microbiome OR microbioma gastrointestinal OR microbiom$ OR microbiota OR ecosystem OR ­*ecossistema* OR *ecosistema*) OR (probiotic$ OR bifidobacterium OR lactobacillus) AND (constipation OR *constipação* OR *estreñimiento*). References to articles and other relevant systematic reviews were also consulted.

After reading the titles and abstracts, we selected all original articles that included children and adolescents. To evaluate fecal microbiota in constipation, the articles that compared children and adolescents with constipation and a control group were considered. Inclusion criteria for articles evaluating the use of probiotics in the treatment of constipation were: evaluating children or adolescents aged ≤19 years; including an intervention with any type of probiotic, regardless of the strain, dose and presentation. Research was not restricted to the exclusive evaluation of randomized controlled trials. This systematic review did not include review articles, editorials or commentaries.

## RESULTS

**Figure 1:**
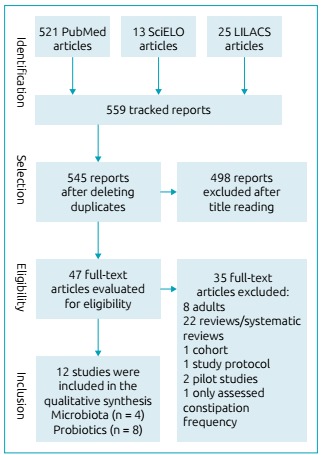
Study flowchart.

At the beginning of the research on microbiota, probiotics and constipation, 559 articles were found, of which 47 were selected for reading. Of these, 12 articles that studied children and adolescents were included in this review.^8^ ^,^ ^9^ ^,^ ^13^ ^,^ ^15^ ^,^ ^16^ ^,^ ^17^ ^,^ ^18^ ^,^ ^19^ ^,^ ^20^ ^,^ ^21^ ^,^ ^22^ ^,^ ^23^ According to their content, the articles were divided into two categories: evaluation of the relationship between fecal microbiota and constipation; and the use of probiotics in the treatment of functional constipation. Figure 1 shows the study’s flowchart.

Thus, four articles evaluated changes in fecal microbiota in constipation in children and adolescents.^8^ ^,^ ^9^ ^,^ ^13^ ^,^ ^15^ These studies were performed in Italy,^8^ the United States,^9^ Holland^13^ and Brazil,^15^ and included between 22 and 137 individuals. The methods used to analyze the fecal microbiota were different in each study. The summary of the information in these articles is presented in Table 1.

**Table 1:** Characteristics of studies evaluating the gut microbiota of children and adolescents with constipation.

Authors, year, reference, location	n (age) division of groups	Definition of constipation	Analysis of fecal microbiota	Results of children with constipation compared to controls
Zoppi et al., 1998^8^ Italy	42 (5-14 years old) 28 with constipation 14 healthy controls	Bowel movement frequency: <1 every 48 hours and hard stools	Material analyzed: a stool sample. Culture in selective and non-selective media supplemented by biochemical assay	- Similar anaerobic total count in individuals with and without constipation. In those with constipation, we observed: - a higher number of anaerobes of the genera *Clostridia* (p <0.001) and *Bifidobacteria* (p <0.02); - a greater number of *Clostridium* than *Bacterioides*; - a greater number of *Clostridium* than *Escherichia coli*. In the control group, the counts of *Clostridia*, *Bacteroides* and *E. coli* were similar.
Zhu et al., 2014 ^9^ United States	22 (10-13 years old) eight obese patients with constipation 14 obese controls without constipation	NASPGHAN Guideline (2006):^39^ delay or difficulty having a bowel movement for more than two weeks	Material analyzed: a stool sample. Pyrosequencing of the 16S rRNA gene	In those with constipation, we observed: - a decrease in the filament *Bacteroidetes*, especially genus *Prevotella* (0.010); - an increase in *Firmicutes*, especially *Lachnospiraceae* (p = 0.042) and *Ruminococcaceae* (p = 0.024); Constipation was not associated with a decrease in *Lactobacillus* and *Bifidobacteria*.
Meij et al., 2016 ^13^ Holland	137 (4-18 years old) 76 with constipation 61 healthy controls	Rome III Criterion (2006) for functional constipation	Material analyzed: a stool sample. Microbiota profile based on IS-pro multiplex PCR method	- No differences were found in phyla and bacterial diversity according to Shannon’s index. In those with constipation, the following were found: - an increase in *Bacteroides fragilis*, *Bacteroides ovatus*, *Bifidobacterium longum*, *Proteus mirabilis*, a species of *Parabacteroides*; - a decrease in *Alistipes finegoldii* and*Ruminococcus* species.
Moraes et al., 2016 ^15^ Brazil	79 (3-36 months) 39 with constipation 40 healthy controls	Rome III Criterion (2006) for functional constipation	Material analyzed: a stool sample. Total Bacterial Count, *Lactobacillus* and *Bifidobacterium* by real time PCR	- No difference was observed in the counts for total bacterial and bifidobacterium. - In those with constipation, a lower (p = 0.022) concentration of *Lactobacillus* per milligram of stool was observed.

NASPGHAN: North American Society for Pediatric Gastroenterology, Hepatology and Nutrition.

Zoppi et al.^8^, using conventional culture techniques, compared 28 children that had constipation with 14 controls, and observed a statistically significant increase in the *Clostridium* and *Bifidobacterium* genres.

In the study performed by Zhu et al.,^9^ using 16S rRNA gene pyrosequencing, the fecal microbiota of eight children with constipation and obesity were compared with 14 obese controls. The authors observed that the abundance of *Bacteroidetes* is lower in constipation (mainly species of the *Prevotella* genus), while several families and genus of the *Firmicutes* phylum were in greater abundance.

Meij et al.^13^ used a technique called IS-pro for multiplex PCR (polymerase chain reaction), and observed a greater abundance of bifidobacteria (*Bifidobacterium longum*, *B. fragilis* and *B. ­ovatus*) in individuals with constipation. Fecal microbiota analysis could differentiate between the 76 patients with constipation and the 61 healthy controls with 82% accuracy.

In the study performed by Moraes et al.,^15^ using real-time PCR to search for *Lactobacillus* and *Bifidobacterium*, a lower concentration of *Lactobacillus* was found per milligram of feces in children with constipation.

The use of probiotics in the treatment of functional constipation has been evaluated in eight articles,^16^ ^,^ ^17^ ^,^ ^18^ ^,^ ^19^ ^,^ ^20^ ^,^ ^21^ ^,^ ^22^ ^,^ ^23^ which were performed in Italy,^16^ ^,^ ^17^ Poland,^18^ ^,^ ^19^ Holland and Poland,^20^ Taiwan,^21^ Brazil^22^ and Iran.^23^ The main features of the studies are shown in Table 2.

**Table 2:** Characteristics of placebo-controlled randomized trials of probiotics / comparison in constipation in children and adolescents.

Study, year, reference	Patients		Probiotic		Comparison (n)/presentation	Duration	Allocation concealment/blind analysis/intention to treat/follow-up losses
	n (age)	Definition of constipation	Genus, species and strain (n)	Dose/presentation			
Banaszkiewicz et al., 2005^18^	(2-16 years old)	Less than three bowel movements/wk. for at least 12 weeks	*L. rhamnosus GG* ATCC 531032 + Lactulose (n=43)	2x/day 10 ^9^ CFU/capsules	placebo + lactulose (n = 41)/capsules	26 weeks (12 weeks of treatment)	yes/yes/yes/yes
Bu et al., 2007^21^	45 (1-4 years old)	Less than three bowel movements/wk. for more than two months and anal fissure with bleeding or faecal leak or hard/large stools	*L. casei rhamnosus* Lcr35 (n = 18)	2x/day 8 × 10^8^ CFU/capsules	MgO (n = 18) Placebo (n = 9)/capsules	4 weeks	yes/yes/yes/yes
Coccorullo et al., 2010^16^	44 (5-10 months old)	Rome III Criterion (2006) for functional constipation	*Lactobacillus* *reuteri* DSM 17938 (n = 22)	1x/day 10^8^ CFU/oily suspension in drops	Placebo (n = 22)/oily suspension in drops	eight weeks	yes/yes/yes/yes
Tabbers et al., 2011^20^	148 (3-16 years old)	Rome III Criterion (2006) for functional constipation	*Bifidobacterium lactis* DN-173 010 (n=74)	2x/day 4.25 x 10^9^ CFU/fermented milk	Placebo (n=74)/Low lactose non-fermented dairy	five weeks (three weeks of treatment)	yes/yes/yes/yes
Guerra et al., 2011^22^	59 (5-15 years old)	Rome III Criterion (2006) for functional constipation	*Bifidobacterium longum* (n = 29)	1x/day 10^9^ CFU/goat yogurt	Placebo (n = 30)/goat yogurt	ten weeks (crossover after five weeks)	yes/yes/yes/yes
Sadeghzadeh et al., 2014^23^	48 (4-12 years old)	Rome III Criterion (2006) for functional constipation	Protexin^*^ (n = 24)	1x/day 10^9^ CFU + lactulose/sachet	placebo + lactulose (n = 24)/sachet	four weeks	yes/yes/yes/yes
Russo et al., 2017^17^	55 (4-12 years old)	Rome III Criterion (2006) for functional constipation	Probiotic Mix^**^ + PEG 4000 (n = 27)	1 sachet/day	PEG 4000 (n = 28)/sachet	eight weeks	yes/no/yes/yes
Wojtyniak et al., 2017^19^	81 (1-4 years old)	Rome III Criterion (2006) for functional constipation	*Lactobacillus casei rhamnosus* Lcr35 (n=46)	2x/day 8 × 10 8 CFU/capsules	Placebo (n = 48)/capsules	four weeks	yes/yes/yes/yes

CFU: colony forming units; PEG: polyethylene glycol (0.4 to 0.8 g/kg/day); MgO: magnesium oxide (50 mg/kg/day); lactulose (1 ­mL/­kg/­day); ^*^Protexin^®^: *Lactobacillus casei* PXN 37, *Lactobacillus rhamnosus* PXN 54, *Streptococcus thermophiles* PXN 66, *Brief bifidobacterium* PXN 25, *Lactobacillus acidophilus* PXN 35, *Bifidobacterium infantis* PXN 27 and *Lactobacillus bulgaricus* PXN 39; ^**^probiotic mix: *Brief bifidobacterium* M-16 V^®^, *Infant Bifidobacterium* M-63^®^ and *Bifidobacterium longum* BB536^®^.

The probiotic strains used for the intervention were predominantly from the genera *Bifidobacterium* and *Lactobacillus*: *B. lactis* DN-173010,^20^ *B. longum*,^22^ *L. reuteri* DSM 17938,^16^ *L. casei rhamnosus* Lcr35,^19^ ^,^ ^21^ *LGG* ATCC 531032,^18^ *B. breve* M-16 V^®^, *infantis* M-63^®^, e *longum* BB536^®^ ^17^ and Protexin^®^ (*L. casei* PXN 37, *L. rhamnosus* PXN 54, *S. thermophiles* PXN 66, *B. breve* PXN 25, *L. acidophilus* PXN 35, *B. infantis* PXN 27, e *L. bulgaricus* PXN 39).^23^

Of these articles, only one found a higher frequency of probiotic therapeutic success in the treatment of constipation with statistical significance, using the strain *Lactobacillus casei rhamnosus* Lcr35.^21^ Higher bowel movement frequency,^16^ ^,^ ^21^ ^,^ ^23^ improved stool consistency,^21^ ^,^ ^23^ reduced abdominal pain,^21^ reduced pain during bowel movements ^22^ and flatulence reduction were observed.^20^ The main results are shown in Table 3.

**Table 3:** Results summary of studies evaluating the role of probiotics in the treatment of constipation in children and adolescents.

Author, year, reference and place	Probiotic	Results of children with constipation compared to control group
Banaszkiewicz et al., 2005^18^ Poland	*L. rhamnosus GG* (ATCC 531032)	Treatment success^a^ was similar (p> 0.05) at the 12^th^ week (experimental group=72% and control group=68%) and at the 24^th^ week (experimental group =64% and control group=65%). There was no difference between the groups regarding weekly number of bowel movements, the force of the bowel movement, fecal escape and the number of required doses of laxatives.
Bu et al., 2007^21^ Taiwan	*L. casei rhamnosus* Lcr35	Greater (p=0.01) treatment success^a^ in the magnesium oxide (72.2%) and probiotic (77.8%) groups compared to the placebo (11.1%). The magnesium oxide and probiotic groups presented a higher (p = 0.03) frequency of bowel movement, a lower (p = 0.01) frequency of hardened stools and a lower (p = 0.04) frequency of glycerin enema use in comparison to the placebo. Episodes of abdominal pain were less frequent (p = 0.03) in the probiotic group compared to the magnesium oxide and placebo groups. There was an increase in the percentage of lactobacilli in anaerobic microbiota after probiotic treatment (p = 0.03) and when compared to the magnesium oxide and placebo groups (p = 0.02), there was no correlation with bowel movement frequency. There was no difference between the groups regarding the frequency of lactulose use, bowel movement episodes and appetite alteration.
Coccorullo et al., 2010^16^ Italy	*Lactobacillus* *reuteri* DSM 17938	No definition of therapeutic success. Higher frequency of probiotic bowel movements in the second (p = 0.042), fourth (p = 0.008) and eighth (p = 0.027) weeks of treatment versus the control. There was no difference between groups with regard to stool consistency. There was an increase (p = 0.02) of inconsolable crying episodes in the probiotic group. In the control group, an increase in inconsolable crying was also observed, however, it did not reach statistical significance (p = 0.08).
Tabbers et al., 2011^20^ Holland and Poland	*Bifidobacterium lactis* DN-173 010 (n=74)	Treatment success^b^ was higher in the probiotic group (38%) compared to the placebo group (24%), but there was no significant difference (p=0.06). In the probiotic group, a reduction (p = 0.02) in flatulence frequency was observed. There was no difference between the probiotic and control groups regarding bowel movement frequency, stool consistency, fecal incontinence, pain during bowel movements, abdominal pain and bisacodil use. Higher bisacodil intake was observed in the control group (p=0.0069).
Guerra et al., 2011^22^ Brazil	*Bifidobacterium longum* (n = 29)	They do not present total data obtained in the two intervention periods with probiotic or control. They mention that in the probiotic group, considering all the results, there was a significant difference in the frequency of bowel movements, pain in bowel movements and abdominal pain.
Sadeghzadeh et al., 2014^23^ Iran	Protexin^®^	No definition of therapeutic success. At the end of the fourth week it was found that the probiotic group had a higher (p = 0.042) bowel movement frequency and an improvement (p = 0.049) in stool consistency when compared to the placebo group. In the first week of intervention, a lower (p=0.030) frequency of fecal incontinence, a lower frequency (p=0.017) of abdominal pain and a greater weight gain (p=0.002) was found. These variables were similar in the fourth week of the study.
Russo et al., 2017^17^ Italy	Brief bifidobacterium M-16 V^®^ , Infant Bifidobacterium M-63^®^ and Bifidobacterium longum BB536^®^. *Bifidobacterium* l*ongum* BB536^®^	In the second week of the study, treatment success^c^ was higher with PEG (72%) compared to the PEG + probiotic mixture group (59%) (p=0.02). After one month (the fourth week), there was no difference in treatment success between the PEG group (88%) and the PEG + probiotic mixture group (81.8%). There was no difference between groups regarding bowel movement frequency, stool consistency, abdominal pain, fecal incontinence and rectal bleeding after two months (eighth week) of study. One month after the end of the study (12^nd^ week), a clinical remission rate was observed in the PEG + probiotic mixture group in 64% of patients and 52% in the PEG-only group (p=0.28).
Wojtyniak et al., 2017^19^ Poland	*Lactobacillus casei rhamnosus* Lcr35	There was no difference in treatment success^a^ between the groups. In the probiotic group, there was a lower (p = 0.005) bowel movement frequency in allof the studied weeks compared to the placebo group. Comparing from the baseline to the fourth week of study, there was an increase (p<0.001) in bowel movement frequency and an improvement (p<0.001) in stool consistency in both groups.

PEG: polyethylene glycol; ^a^treatment success defined as> 3 spontaneous bowel movements per week without fecal leaks; ^b^treatment success defined as >3 bowel movements/week, <1 episode of fecal incontinence in the last two weeks of product consumption; ^c^treatment success defined as ≥3 bowel movements per week; stool consistency ≥ type 3 according to the Bristol scale; and no episodes of abdominal pain, fecal incontinence, painful bowel movement or rectal bleeding.

## DISCUSSION

### Microbiota of children with constipation

Despite the rapid growth of knowledge and scientific literature on gut microbiota and probiotics, the present systematic review has shown that information is very limited regarding the profile of gut microbiota in children and adolescents with constipation. No systematic review on this topic was found.

As can be seen from Table 1, the four articles that evaluated gut microbiota in children with constipation used different analysis methods. Thus, it is difficult to compare the differences found in the gut microbiota in each of the studies.^24^

It is evident that the results obtained on the fecal microbiota of children and adolescents with constipation are discrepant. The different methodologies used for the fecal microbiota analysis of the studied individuals and the different species of bacteria evaluated made it impossible to determine a specific pattern of fecal microbiota abnormalities in patients with constipation.

Regarding the study conducted in the United States,^9^ it should be noted that patients that did and did not have constipation were evaluated, however, all were obese. It should be noted that obesity alone is associated with changes in gut microbiota.^13^ ^,^ ^25^ ^,^ ^26^

Other aspects that hinder the comparability of the obtained results are the high inter-individual variability of the fecal microbiota and the clinical heterogeneity of the studied populations.^13^ ^,^ ^27^ Another factor is the age of the patients studied, considering that children under the age of three are in the microbiota implantation phase, which could interfere with the results.^28^

Therefore, further studies with larger and more homogeneous series, preferably using modern molecular biology techniques, are needed to establish the gut microbiota profile of children and adolescents with constipation, more accurately.

### The use of probiotics for the treatment of constipation in children and adolescents

According to the literature, there are several mechanisms that can potentially explain the action of probiotics in the treatment of functional constipation: modification of the gut microbiota, considering the changes that have been shown in individuals with constipation; increased production of lactate and short chain fatty acids, reducing luminal pH, which could improve colonic peristalsis and decrease intestinal transit time.^8^ ^,^ ^29^ ^,^ ^30^ ^,^ ^31^ Taking this into consideration, there is a growing interest in using probiotics in the treatment of constipation.

The definition of therapeutic success is an important criterion when evaluating the effectiveness of therapeutic interventions. In children and adolescents with constipation, it is important to evaluate not only bowel movement frequency but also stool consistency as parameters for therapeutic success.^32^ As described in Table 3, among the clinical trials included in this systematic review, only one included stool consistency as part of the definition of therapeutic success.^17^ In some of the articles, therapeutic success was not defined and different variables were evaluated separately.^16^ ^,^ ^22^ ^,^ ^23^

As can be seen from Table 2, three studies performed interventions with a laxative-associated probiotic: polyethylene glycol^17^ and lactulose.^18^ ^,^ ^23^ However, these medications are, by themselves, effective therapeutic interventions for the treatment of constipation^1^ ^,^ ^4^ ^,^ ^5^ and their association with probiotics can be important to note, because the therapeutic role of probiotics may be covered up. Furthermore, it was difficult to compare studies in relation to the duration of constipation and the use of laxatives, which may interfere with the success of treatment with probiotics. Thus, in future studies, this type of design should be avoided.

Among the clinical trials in which probiotics without laxatives were used, only one study found greater therapeutic success from the probiotic when compared with the placebo.^21^ In this article, it was further observed that the effect of the probiotic *L. casei rhamnosus* Lcr35 was similar to that of magnesium oxide administered in a third group of the clinical trial (Table 3).

In Table 3, we can also see that irregular beneficial effects of certain probiotics were found in some clinical manifestations of constipation, which resulted in increased bowel movement frequency, ^16^ ^,^ ^21^ ^,^ ^23^ improved stool consistency,^21^ ^,^ ^22^ ^,^ ^23^ reduced abdominal pain,^21^ reduced pain from bowel movements ^22^ and reduced flatulence. ^20^

In these intervention studies using probiotics, the composition of the intestinal microbiota before and after probiotic administration was not evaluated. Thus, the observed clinical effects on motility and characteristics of intestinal habit attributed to the use of probiotics could not be associated with specific changes in the composition of the gut microbiota.

Regarding the methodological quality of the analyzed clinical trials, it is worth noting that the only randomized crossover clinical trial ^22^ did not mention a period of ­*wash-out* between the intersection of the interventions. Thus, in the second stage of the study, there could have been residual effects of the probiotics. It is also important to report that several studies did not have an adequate sample size, as they were composed of convenience samples with a small number of participants, which makes comparison and the reliability of the data obtained, difficult.

Thus, it was found that the intervention studies with probiotics included in this systematic review are heterogeneous with regard to the population studied, the probiotic strains used, the dosages used for treatment, the duration of the study, the follow-up, the definitions, and the parameters used to evaluate the effect of intervention on the control of constipation in children and adolescents. Therefore, the data obtained do not allow for specific recommendations to be made regarding the use of probiotics in the treatment of functional constipation.

In the literature, five systematic review articles were found,^30^ ^,^ ^33^ ^,^ ^34^ ^,^ ^35^ ^,^ ^36^ Two had a meta-analysis, ^30^ ^,^ ^34^ which addressed the use of probiotics in the treatment of functional constipation in children and adolescents. An article published in 2010^33^ included two clinical trials in children^18^ ^,^ ^21^ and three adult trials, and concluded that only *L. rhamnosus* Lcr35 showed a beneficial effect for the treatment of constipation in children. A systematic review and meta-analysis published in 2014^34^ evaluated the use of probiotics for pediatric functional gastrointestinal disorders. Probiotics *LGG*, *L. reuteri* DSM 17 938 and VSL # 3^®^ have been reported to have a better effect than placebo in the treatment of functional abdominal pain and irritable bowel syndrome. However, no evidence was found indicating the efficacy of probiotics in the treatment of functional constipation. A systematic review published in 2016 ^35^ on the use of prebiotics, probiotics and symbiotics for the treatment of functional constipation in children concluded that there is still insufficient evidence to support the recommendation of probiotics in this treatment. At the same time as the present article was being completed, two systematic reviews were published^30^ ^,^ ^36^ addressing this theme. In 2017, a systematic meta-analysis review highlighted the effectiveness of probiotics for improving bowel movement frequency in Asian children, and highlighted the heterogeneity of the studies.^30^ Another systematic review, published that same year, included studies published up until February 2017, and evaluated seven clinical trials.^16^ ^,^ ^18^ ^,^ ^19^ ^,^ ^20^ ^,^ ^21^ ^,^ ^22^ ^,^ ^23^ It was noted that some probiotic strains had some effects on the frequency of bowel movements, but no effects on the frequency of fecal incontinence and abdominal pain.^36^

Given the heterogeneity of clinical trials conducted with children and adolescents, there is a need to standardize the criteria and definitions used to compare the effects of different therapeutic interventions, as mentioned in the literature.^37^ ^,^ ^38^ Therefore, regardless of the analyzes performed, there is not enough evidence to support the recommendation of probiotics for the treatment of constipation in children and adolescents. The ideal approach and treatment of fecal disimpaction is the use of oral medication and the education of family members and patients^39^.

It was not possible to determine a specific pattern of fecal microbiota abnormalities of constipation in children and adolescents.

Irregular beneficial effects of probiotics were evidenced in some clinical manifestations of constipation in this population. However, clinical trials are still scarce and heterogeneous, and their results are controversial.

To date, there is no scientific evidence to support probiotic supplementation for the treatment of functional constipation in children and adolescents.

Thus, further research with well-established and homogeneous methodologies is needed to determine causal relationships between fecal microbiota alteration and constipation, as well as on the effectiveness of using probiotics to treat children and adolescents
